# Perception Mechanism of Bone-Conducted Ultrasound and Its Clinical Use

**DOI:** 10.3390/audiolres11020022

**Published:** 2021-05-30

**Authors:** Tadashi Nishimura, Tadao Okayasu, Akinori Yamashita, Hiroshi Hosoi, Tadashi Kitahara

**Affiliations:** 1 Department of Otolaryngology-Head and Neck Surgery, Nara Medical University, 840 Shijo-cho, Kashihara, Nara 634-8522, Japan; tokayasu@naramed-u.ac.jp (T.O.); akinori@naramed-u.ac.jp (A.Y.); tkitahara@naramed-u.ac.jp (T.K.); 2 MBT (Medicine-Based Town) Institute, Nara Medical University, 840 Shijo-cho, Kashihara, Nara 634-8522, Japan; hosoi@naramed-u.ac.jp

**Keywords:** bone conduction, ultrasound, ultrasonic perception, high frequency sound, profound deaf, tinnitus

## Abstract

It is generally believed that ultrasound cannot be heard. However, ultrasound is audible when it is presented through bone conduction. Bone-conducted ultrasound (BCU) has unique characteristics; the most interesting is its perception in patients with profound deafness. Some patients can perceive it and discriminate speech-modulated BCU. Previous reports have suggested that BCU can be used for a hearing aid or tinnitus sound therapy. In this review, the perception of BCU at both the peripheral and central levels was investigated based on previous studies, although some of them remain controversial. We also investigated the clinical use of BCU. To develop hearing aids utilizing BCU, the encoding of speech signals into BCU has to be established. The outcomes of the reported speech modulations were evaluated. Furthermore, the suppression of tinnitus by BCU was reviewed, and the feasibility of the application of BCU to tinnitus treatment was investigated.

## 1. Introduction

The audible frequency range of the human ear is between 16 Hz and 24 kHz [1], and a sound above this frequency range is referred to as “ultrasound.” In contrast, ultrasound, whose frequency ranges up to at least 120 kHz, can create an auditory sensation when delivered via bone conduction (BC) [2,3,4]. Previous studies indicated that middle ear impedance might prevent ultrasound transmission via air conduction (AC) [3,5]. Other previous studies have suggested the generation of audible sound due to a nonlinear process in BC [4,6]. If a generated audible sound is predominantly associated with the perception of bone-conducted ultrasound (BCU), the characteristics of the induced sensation should resemble those of the audible sound. However, the reported characteristics of BCU perception (ultrasonic perception) are unique and not always observed with the perception of air-conducted audible sound (ACAS). In this review, ACAS was defined as an audible sound to avoid confusion in terminology. “Audible sound” was used for ACAS and distinguished from “ultrasound,” although ultrasound is audible via BC.

## 2. Characteristics of Ultrasonic Perception

According to previous reports, the pitch of BCU resembles that of ACAS at frequencies of 8–16 kHz, independent of its own frequencies [3,4,6]. The dynamic range is very narrow and similar to that obtained in patients with a cochlear implant [7]. The most interesting unique characteristic is the perception in patients with profound deafness. Some of them can perceive BCU [8,9,10], which suggests the contribution of a unique perception mechanism different from that of ACAS. Lenhart et al. found that some patients with severe hearing loss could discriminate BCU stimuli modulated with speech [8]. Cochlear implants are usually required in patients with profound deafness [11,12]. If a speech signal is delivered with an ultrasonic hearing device, a profoundly deaf individual can recover speech perception without any surgical operation. To apply this method in clinical practice, the mechanism underlying its perception should be established.

## 3. Peripheral Perception Mechanism of BCU

For audible sound, the presentation of an acoustic stimulus can disturb the perception of other sounds (masking), and the amount of masking grows as the difference between the frequencies of the two sounds reduces. The masking of ACAS by BCU can be used to establish its perception mechanism. In our previous study, ACAS at frequencies below 8 kHz was not masked by BCU, which suggested that ultrasonic perception was not significantly associated with the perception of ACAS at frequencies of ≤8 kHz [7]. In contrast, the threshold of high-frequency ACAS (above 8 kHz) was remarkably elevated under a low-sensation level (SL) BCU presentation. With a 5-dB SL BCU presentation, the maximum amount of the masking was recognized at approximately 30 dB at 14 kHz. The peak of the masking patterns occurred between 10 and 14 kHz, which was independent of the ultrasound frequency [7]. Furthermore, the increase in BCU masker to 10 dB SL induced greater masking. Masking increases faster than linearly, showing a rate of 2–5.8 dB/dB between 9 and 15 kHz, and the maximum growth was observed at 10 kHz. For ACAS, an increase in masking of more than 1 dB/1 dB can be observed when the masker frequency is lower than that of the signal [13]. If the current masking were produced by a demodulated sound, the characteristic frequency of the modulated sound would be below 10 kHz. However, it was lower than the peak of masking patterns. Thus, it appears that the observed masking was not produced by the demodulated sound but by the ultrasound itself. Nakagawa et al. measured the vibration of the eardrum with a laser Doppler when ultrasound was presented using BC [14]. No significant generation of the demodulated audible sound was identified, and they concluded that nonlinear distortion does not contribute to perception. Considering the responsivity of the outer hair cells (OHCs) to the ultrasonic frequency range, the active process derived from OHCs cannot probably function during ultrasonic perception. According to Bekesy’s traveling wave theory [15], ultrasound causes the largest vibration amplitude at locations basal to those responding maximally to high-frequency ACAS. If the theory were applied to the vibrations of the basilar membrane produced by BCU, they would become stronger and spread downward as intensity increases, which probably produces both masking of high-frequency ACAS and generation of auditory sensation [7]. 

**Figure 1:**
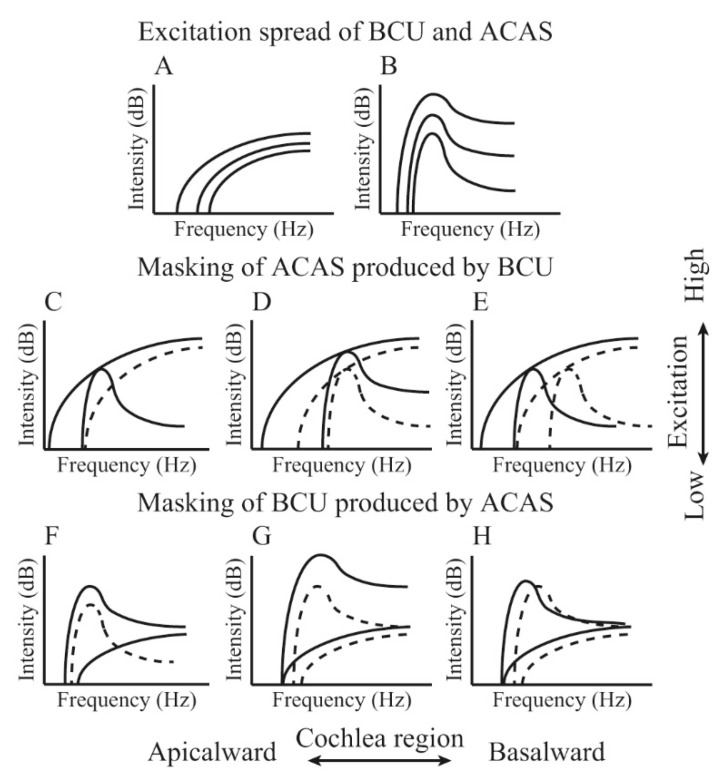
Schema of the excitation pattern and growth of masking by bone-conducted ultrasound (BCU) and air-conducted audible sound (ACAS). Parts (**A**,**B**) indicate the excitation pattern depending on the intensity. For BCU, the excitation does not show a sharp peak and spreads fast to the apical ward (Part (**A**)). For ACAS, the excitation pattern shows a peak at the characteristic frequency and spreads slowly to the apical ward (Part (**B**)). Part (**C**) indicates masking of ACAS by BCU. ACAS cannot be masked with a low-level masker (dashed line). However, when the excitation by BCU covers that by ACAS (solid line), ACAS is masked. Parts (**D**,**E**) indicate the growth of masking depending on the intensity. The dashed and solid lines indicate the masking pattern observed at a low- or high-level masker, respectively. The masking grows fast (Part (**D**)) and also spreads fast to the apical ward (Part (**E**)). In contrast, Part (**F**) indicates masking of BCU by ACAS. At low ACAS masker intensity (dashed line), the excitation pattern shows a sharp peak. Because BCU excites a broad cochlear region, ACAS below 60 dB SPL cannot mask BCU. When the masker intensity is sufficiently large (solid line), the excitation by ACAS covers a broad cochlear region and masks BCU. Parts (**G**,**H**) indicate the growth of masking, depending on the intensity. The dashed and solid lines indicate the masking at low and high signal intensities, respectively. The excitation by BCU fast spreads to the apical ward. Accordingly, a large increase in masker intensity (Part (**G**)) or masker frequency shift to the lower frequency range (Part (**H**)) is required for masking. In each schema, vertical and horizontal axes indicate the excitation and cochlear region, respectively. (Figure 1 was originally presented in Nishimura et al. 2011, Figure 1 [17]).

If the peripheral perception organ of BCU is located in the cochlea, ultrasonic perception may be possible to be completely masked by ACAS. In previous studies, ultrasonic perception was barely masked by ACAS [4,16]. However, given the masking produced by BCU, ultrasonic perception is most likely associated with high-frequency ACAS perception [7]. If the masking spectrum does not sufficiently cover its frequency range, the ultrasonic perception cannot be masked. BCU can excite a broad cochlear region, considering the masking pattern of ACAS produced by BCU [7]. When the intensity of the ACAS masker is sufficiently high, the excitation spreads broadly depending on the intensity. An ACAS that excites a sufficient cochlear region can mask the ultrasonic perception. Our previous study found that ultrasonic perception was masked by high-frequency ACAS, especially when the masker frequency and intensity were 10–14 kHz and above 80 dB SPL, respectively [17]. The frequency of an ACAS masker, which could mask ultrasonic perception, was equal to that of an ACAS, which was strongly masked by BCU [7]. Consequently, it is suggested that ultrasonic perception is associated with a high-frequency ACAS perception, and both perceptions may have common auditory pathways. Figure 1 shows a schematic of the mechanism of ultrasonic perception and masking. Our studies on masking produced by BCU and masking of ultrasonic perception by ACAS corroborated our hypothesis.

In our hypothesis of the peripheral perception mechanism, the function of OHCs does not contribute to ultrasonic perception. Inner hair cell (IHC) activity induced by ultrasound plays an important role in ultrasonic perception, even without the presence of modulation, and it does not depend on the enhancement of BCU by OHCs in the basal turn of the cochlea. Regarding the thresholds of BCU in patients with hearing impairment, no patients with an estimated total loss of the IHC system in the cochlear basal turn could hear BCU [17]. A significant relationship was found between BCU thresholds and the estimated extent of the presence of IHC function in the cochlea. In another study, the impact of cisplatin administration on the thresholds of ACAS and BCU was evaluated in patients with head and neck cancer [18]. Ototoxic drugs, such as cisplatin usually induce hearing deficits predominantly due to the disorder of OHCs [19]. The damage to OHCs can induce the threshold elevations of ACAS, and the thresholds of BCU can be maintained owing to the independence of the OHC function. In the results, although the thresholds for high-frequency ACAS significantly worsened after treatment, the thresholds of BCU did not increase [18]. These findings are consistent with our hypothesis for the perception mechanisms. With the findings in the previous studies, the peripheral perception mechanism has been gradually revealed but remains controversial.

## 4. Ultrasonic Perception at the Central Level

In previous studies on ultrasonic perception at the central level, neural activation was observed with magnetoencephalography and positron emission tomography in the auditory cortex but not in the somatosensory cortex [9,10]. These findings objectively established that ultrasonic perception is an auditory sensation, not a somatosensory sensation. Previous studies evaluated N1m responses evoked by BCU and compared them with those for ACAS [20,21,22,23,24,25]. These studies demonstrated several differences between the characteristics of ACAS and BCU, suggesting a unique mechanism underlying the perception of BCU at the central level. The magnitude of N1m grows as a function of intensity and duration [21,24]. The growth rates of N1m amplitude are differed for ACAS and BCU, which may be associated with a narrow dynamic range of ultrasonic perception. However, the growth of the N1m amplitude was saturated approximately at the uncomfortable loudness level or the duration of 40 ms for both stimuli [22,24]. Regarding the detection of the frequency difference, the mismatch field (MMF) evoked by the frequency change for BCU was significantly lower than that for ACAS [23]. Considering the peripheral mechanism, excited cochlear regions depend on intensity but not frequency [17]. Therefore, distinguishing the frequency of BCU is difficult. The unique characteristics of the central auditory response for BCU may be explained by the differences in the peripheral perception mechanism. The fundamental neural system at the central level is probably similar to that of ACAS. Further studies are required for the establishment of ultrasonic perception at the central level as well as the peripheral.

## 5. Clinical Use of Ultrasonic Perception

One of the clinical applications of ultrasonic perception is hearing aids for profound deafness. Some characteristics of ultrasonic perception resemble those of the perception provided with a cochlear implant rather than those of ACAS perception. The most pertinent difference between BCU and cochlear implants is the spectral resolution. BCU can stimulate one broad cochlear region in the basal turn, which can be considered a single-channel hearing device, whereas cochlear implants have multiple channels [26,27,28]. Speech has to be encoded by ultrasound for communication using this single channel. The role of the temporal fine structure and envelope is important for the perception of speech [29,30]. Previous studies employed amplification modulation as the encoding method [31,32,33]. During the encoding process, speech is used as a carrier sound, and BCU is modulated with this carrier. The envelope of the speech-modulated BCU can transmit cues for speech recognition.

## 6. Recognition of Speech-Modulated BCU in Normal-Hearing Individuals

In previous studies, speech-modulated BCU facilitated good speech recognition scores for participants with normal hearing [31,32,33]. These results are interesting because the speech-modulated BCU was accurately recognized without any training. Demodulation in the nonlinear process is probably associated with high recognition scores [34]. Fujimoto et al. evaluated the difference limens for frequency of BCU modulated by air-conducted pure tones under two conditions: using amplitude modulation based on a double-side-band transmitted carrier and a suppressed carrier [35]. Comparing the pitches of these conditions, they demonstrated a nonlinear process underlying the perception of modulated BCU.

Changes in voice pitch contribute to communication. During conversation, speech sounds convey both linguistic and non-linguistic information, such as attitudes and emotions. Prosody is important for the conveyance of information, such as questions or affirmations, through speech and the expression of emotions. In a previous behavioral study, prosodic information for speech-modulated BCU could also be distinguished during normal hearing [36]. Furthermore, prosodic discrimination for speech-modulated BCU was objectively evaluated by measuring the MMFs elicited by prosodic and segmental changes [37]. In almost all the participants, prominent MMF was elicited by prosodic and segmental changes in speech-modulated BCU. Although the discrimination of speech-modulated BCU is slightly inferior to that of air-conducted speech, prosodic change can be distinguished to the same degree as segmental change, even for speech-modulated BCU.

## 7. Recognition of Speech-Modulated BCU in Hearing-Impaired Patients

If demodulation works in the perception of speech modulated BCU, good speech recognition without any training by participants with normal hearing can be explained without difficulty. BCU modulated by speech signals may be demodulated into original signals, and, consequently, most participants would be able to recognize them. In contrast, this also means that hearing-impaired patients, especially profoundly deaf patients, cannot recognize any speech signals because demodulated audible sound cannot be recognized because of their hearing disorder. In contrast, Lenhart et al. reported the discrimination of speech-modulated BCU in patients with severe hearing loss [8]. Hosoi objectively demonstrated the perception of speech-modulated BCU in hearing-impaired patients using MEG [9]. Regarding N1m evoked by speech-modulated BCU in individuals with normal hearing, the impact of the duration of speech-modulated BCU on the N1m amplitude and latency was different from that of AC speech [38]. These results suggest that N1m evoked by speech-modulated BCU is influenced mainly by the ultrasonic component as well as the demodulated audible sound. Both the signal and carrier are associated with the perception, and the discrimination cue of the original speech signal is obtained from the demodulated signal in individuals with normal hearing. By suppressing the perception of demodulated sounds, the responses to the BCU components may be evaluated. Since BCU is difficult to mask by ACAS with a frequency of ≤8 kHz [7], demodulated sound can be masked by an ACAS masker with the minimum effect of ultrasonic perception. The results obtained can be utilized for the development of hearing devices in patients with hearing impairment. 

**Figure 2:**
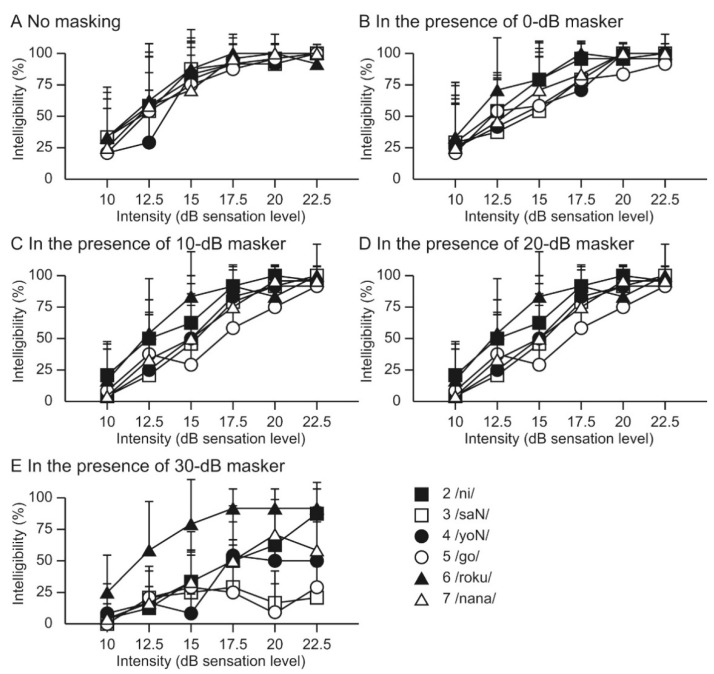
The average scores for the correct answers for each masker condition. The intelligibility of speech-modulated 30 kHz BCU was measured under five masking conditions in eight normal hearing volunteers. A numeral word list was used as speech materials. The ultrasonic transducer was fixed on the forehead. A speech-weighted noise was employed as masking and binaurally presented using earphones. The scores decreased as the masker intensity increased. The reduction in the scores showed variations among the stimuli. The vertical bars indicate standard deviations. (Figure 2 was originally presented in Nishimura et al. 2014, Figure 6 [39]).

**Figure 3:**
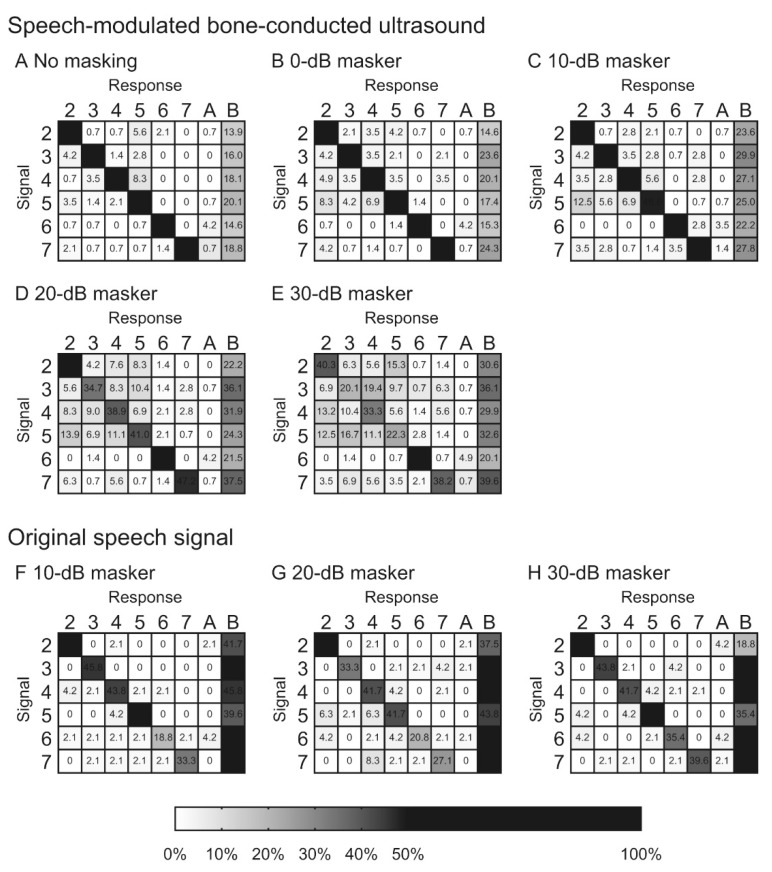
Confusion matrices based on the results of speech-modulated bone-conducted ultrasound (**A**–**E**) and original speech signals (**F**–**H**) under different masking conditions. The results were obtained in the above-mentioned eight normal hearing volunteers. Presentation signals are represented on the left axis and responses across the top axis. “A” and “B” indicate other responses besides the six numeral words and no response, respectively. Blocks with larger grey values (i.e., darker shading) indicate higher appearance frequencies for those pairs. When the appearance frequency was higher than 50%, the block is marked fully black. Numerical values in the cells indicate the percentage of the appearance frequency. (Figure 3 was originally presented in Nishimura et al. 2014, Figure 10 [39]).

Our previous study investigated the intelligibility of speech-modulated BCU using a numeral word list under masking conditions [39]. In the presence of masking, the speech recognition curve for the original speech signal shifted to the right, depending on the masker intensity. In contrast, while masking influences the intelligibility of the speech-modulated BCU, the curve did not shift upward, which was similar to the original speech signal (Figure 2). These differences in the masking effect suggest that the recognition of speech-modulated BCU differs from that of the original speech signal. If recognition is performed solely by demodulation, the recognition curve shifts upward depending on the masking intensity. The difference between the results for speech-modulated BCU and the original speech signal indicated the importance of direct ultrasonic stimulation, particularly under the masking condition. The characteristics of the confusion pattern for speech-modulated BCU were different from those of the original speech signal depending on the masker intensity. The frequency of confusion of some words correlated positively with increasing masker intensity (Figure 3). Under high-intensity masking conditions, the demodulated sound is strongly masked by the speech-weighted noise; therefore, participants are forced to respond based on the unmasked ultrasonic stimulation. The previous findings indicated the difference between speech-modulated BCU and the original speech signal and showed that direct ultrasonic stimulation, as well as demodulated sound, contributed to recognition. However, the current data also suggest that information transmitted solely by ultrasonic stimulation is insufficient to recognize the conversation. A good speech recognition score is probably difficult to obtain with the present BCU device in profoundly deaf patients. To improve the benefits, visual information may facilitate communication with an ultrasonic hearing device [33]. Additional developments are necessary for clinical applications.

## 8. Application of BCU for Tinnitus Treatment

Tinnitus is a common audiological disease, and the prevalence of continuous subjective tinnitus among adults ranges from 5.1% to 42.7% [40]. It negatively affects the quality of life [41], and the prevalence of depressive disorders in this population can be high, ranging from 10% to 90% [42]. However, the efficacy of pharmacological and behavioral interventions remains limited. Sound therapy and psychological approaches have become mainstream for treatment [43,44]. In sound therapy, patients regularly hear sounds using sound applications or sound devices, such as hearing aids, sound masking generators, or modified-sound/Notched-music devices [45]. These sound therapies cannot be conducted in patients with profound deafness due to the severity of their hearing deficits. In contrast, BCU may be utilized for sound therapy in patients with profound deafness because some of them can hear BCU [8,9,10]. In contrast with hearing aids, the predominant aim of sound therapy is the mitigation of the symptoms, not the improvement of speech recognition. Thus, BCU is available more easily for sound therapy in patients with severe hearing loss.

Tinnitus is temporarily masked by presenting a sound, and it is continuously reduced or disappears during the few seconds or minutes after the offset of the masker presentation. This continuous reduction or disappearance of tinnitus is referred to as residual inhibition (RI). RI has been regarded as a clinical index that reflects the degree of tinnitus inhibition [46]. Goldstein et al. found that 20–26 kHz BCU masked tinnitus in 52 patients [47]. They suggested that BCU may be effective in masking tinnitus [48]. However, they did not measure the RI. Koizumi et al. evaluated the RI induced by BCU in 21 patients with tinnitus [49]. The masker intensities of the 30-kHz BCU and audible sounds were set at the minimum masking levels of tinnitus plus 3 dB and 10 dB, respectively. The duration of RI induced by the 30-kHz BCU was significantly longer than that of the RI induced by the 4-kHz sounds. The peripheral stimulation characteristic of BCU probably contributed to inducing long RI durations. Considering the lower presentation of the BCU masker, these findings suggest that BCU suppresses tinnitus more effectively than ACAS.

Sound therapy is regularly administered during daily life. When a sound generation device is used for sound therapy, a generated sound is presented with an earphone for a defined therapy session. During the session, hearing can be disturbed by the generated sound. However, if BCU is used as the presented sound, it rarely affects hearing within the frequency range involved in daily conversation. In addition, ultrasound is delivered via BC, and the insertion of an earphone is not required. Thus, sound therapy utilizing BCU may minimize its influence on daily life and provide good benefits. Further studies on tinnitus therapy using BCU are required.

## 9. Conclusions

BCU is perceived in the basal turn of the cochlea. However, its perception mechanism is different from that of ACAS. The intelligibility for the speech-modulated BCU is comparable to that for the original speech signal in normal-hearing individuals due to the contribution of demodulation. Unfortunately, the lack of the contribution of demodulation to speech perception has to be taken into consideration in hearing impairment patients. The performance of the reported speech-modulated BCU in speech recognition is limited in patients with profound deafness, and further innovation may be required for clinical use. Regarding the application to tinnitus treatment, BCU devices have unique advantages. Unfortunately, the knowledge of BCU in this field has not been sufficient, and further study is required for its establishment.

## Data Availability

Not applicable.
